# Leptin antagonism attenuates hypertension and renal injury in an experimental model of autoimmune disease

**DOI:** 10.1042/CS20230924

**Published:** 2023-12-14

**Authors:** William J. Kalusche, Clinton T. Case, Erin B. Taylor

**Affiliations:** Department of Physiology and Biophysics, University of Mississippi Medical Center, Jackson, MS 39216, U.S.A.

**Keywords:** autoimmunity, hypertension, leptin, lupus, T-cells

## Abstract

Systemic lupus erythematosus (SLE) is a chronic autoimmune disorder that is characterized by B- and T-lymphocyte dysfunction and altered cytokine production, including elevated levels of the adipocytokine leptin. Leptin has various immunomodulatory properties, including promoting the expansion of proinflammatory T lymphocytes and the proliferation and survival of B cells. In the present study, we hypothesized that leptin antagonism would improve B- and T-cell dysfunction and attenuate hypertension in an experimental model of SLE, the NZBWF1 mouse. To test this hypothesis, 28-week-old female control and SLE mice were administered 5 mg/kg of murine leptin superantagonist (LA) or vehicle via ip injection every other day for four weeks. Analysis of peripheral blood immune cell populations showed no changes in total CD45R^+^ B and CD3^+^ T cell percentages after treatment with LA. However, SLE mice treated with LA had an improved CD4/CD8 ratio and decreased CD3^+^CD4^−^CD8^−^ double negative (DN) T cells. Blood pressure was higher in SLE than in control, and treatment with LA decreased blood pressure in SLE mice. Treatment with LA also delayed the onset of albuminuria and decreased glomerulosclerosis in SLE mice. Renal immune cell infiltration was significantly higher in SLE mice as compared with control, but LA treatment was associated with decreased levels of renal CD4^+^ T cells. In conclusion, these data suggest that leptin plays a pathogenic role in the development of hypertension in SLE, in part, by promoting the expansion of inflammatory DN T cells and the infiltration of T cells into the kidneys.

## Introduction

Systemic lupus erythematosus (SLE) is a prototypic multisystem autoimmune disorder that primarily affects women and is most commonly diagnosed during child-bearing years. SLE is characterized by B- and T-cell hyper-reactivity and the production of various autoantibodies, especially to nuclear components. These autoantibodies lead to antigen-autoantibody immune complex formation that deposit in various tissues and lead to the downstream manifestations of the disease, including hypertension, renal injury, and premature development of cardiovascular complications [1–4]. SLE disease is also characterized by imbalances in various cytokines, including those produced by adipose tissue, such as adiponectin, resistin, and leptin [5,6].

Leptin is a 16 kD pleiotropic adipokine that is produced primarily by adipocytes within white adipose tissue. Circulating leptin crosses the blood–brain barrier (BBB) via receptor mediated endocytosis and acts to control appetite and energy expenditure via binding the long form of the leptin receptor (ObRb) on neurons in various regions of the hypothalamus, including the arcuate nucleus, paraventricular nucleus, dorsomedial hypothalamus, ventromedial hypothalamus, and lateral hypothalamus [7,8]. Hypothalamic leptin signaling also plays a role in blood pressure control; leptin’s action within the arcuate nucleus increases sympathetic outflow to the kidney, leading to a rise in arterial pressure [9,10]. In support of this concept, *ob/ob* mice that lack leptin are severely obese yet maintain normal blood pressure [11]. In addition, leptin can act peripherally to impact a range of physiological processes including reproduction, bone formation, carbohydrate and lipid metabolism, intestinal nutrient absorption, and immune function [12].

Leptin receptors are expressed on various cells of both the innate and adaptive immune systems, suggesting that leptin has direct effects on leukocytes and may provide a vital link between nutritional status and immunity [13]. *In vitro* studies have shown that leptin has a proinflammatory function, promotes the secretion of inflammatory cytokines by T helper (Th) Th1 and Th17 cells [14,15], and acts a negative signal for regulatory T-cell proliferation [16]. Studies on B cells indicated that leptin protects them from apoptosis and induces entry into the cell cycle [17], and can stimulate secretion of IL-6, IL-10, and TNF-α [18]. In addition, the CNS actions of leptin impact the sympathetic nervous system (SNS), which is involved in the central control of the immune system [19]. *In vivo* evidence in rodent models indicate that leptin-deficient *ob/ob* mice have impaired host defense against various infections, including *Klebsiella* pneumonia [20], influenza A [21], *Listeria* [22], among many others. In addition, *ob/ob* mice have decreased hematopoietic progenitors in their bone marrow, decreased lymphopoiesis and myelopoiesis, and impaired humoral immune responses [23].

While these studies support an important role for leptin in optimal immune responses, leptin has also been shown to have a detrimental effect on autoimmune disease progression. For example, elevated leptin levels correlate with a reduced number of CD4^+^CD25^+^ regulatory T cells in patients with relapsing-remitting multiple sclerosis, and treatment of mice with a soluble leptin receptor (LepR) fusion protein ameliorates disease in an experimental model of multiple sclerosis [24]. Elevated leptin is also associated with increased disease activity and progression in animal models of autoimmune diabetes [25] and rheumatoid arthritis [26]. When SLE is induced using pristane in *ob/ob* mice, the mice develop lower levels of autoantibodies and exhibit less renal injury [27]. However, the effect of leptin on the development of autoimmune-associated hypertension in SLE is unknown. In the present study, we tested the hypothesis elevated leptin promotes disease pathogenesis, and that antagonism of leptin would improve B and T lymphocyte hyperreactivity and lower blood pressure in the NZBWF1 mouse model of SLE. The NZBWF1 mouse is obese and has elevated leptin levels [28], in addition to developing hypertension and renal injury as the disease progresses [29], thus making it a clinically relevant model of hypertension and obesity in the setting of autoimmune disease.

## Materials and methods

### Animals

Female NZW (*n*=23) and NZBWF1 (*n*=24) mice were used in the present study (Jackson Laboratories, Bar Harbor, ME). Female mice were used because SLE affects women and men at a 9:1 ratio [30], and the NZBWF1 model also has a female preponderance [31]. Mice were maintained on a 12-h light/dark cycle in temperature-controlled rooms (72–75 degrees Fahrenheit) with access to chow (Teklad #8640) and water ad libitum. When mice were 28 weeks of age, they were randomized (*n* = 11–12 mice/group) to receive either vehicle or leptin antagonist (Protein Laboratories Rehovot; Rehovot, Israel) (5 mg/kg every other day) via intraperitoneal injection. The leptin antagonist is a mutated form of mouse leptin containing the amino acid substitutions D23L/L39A/D40A/F41A and monopegylated to enhance *in vivo* activity [32].

Body weight and food intake were assessed daily throughout both studies, and Echo MRI was performed weekly to assess body composition. Mice were also placed in metabolic cages overnight once a week to collect urine for dipstick analysis of urinary albumin (Albustix, Siemens). After 4 weeks of treatment, mice were placed under isoflurane anesthesia (2–3%) and a carotid artery catheter was inserted. The mice were allowed to recover overnight. After two consecutive days of blood pressure measurement, mice were killed using isoflurane overdose followed by cervical dislocation. All studies were performed at the University of Mississippi Medical Center with the approval of the University of Mississippi Medical Center Institutional Animal Care and Use Committee and in accordance with National Institutes of Health Guide for the Care and Use of Laboratory Animals.

### Blood pressure

Mean arterial pressure (MAP, mmHg) was recorded via indwelling carotid artery catheters in freely moving conscious mice as previously described [33–40]. Blood pressure was measured in the morning on two consecutive days using an 8 channel PowerLab/16SP (ADInstruments) blood pressure transducer and recorded using Chart5 for Windows (ADInstruments). A total of 90 min of data were recorded, with the final 60 min being used to determine MAP. The data for the 2 days were averaged for each mouse to determine its MAP.

### Measurement of circulating immunoglobulins and analytes

Total IgM, total IgG, and anti-dsDNA IgG levels in plasma were measured using ELISAs (Alpha Diagnostic) according to the manufacturer’s instructions. Plasma leptin was measured using a mouse/rat leptin ELISA (R&D systems), according to the manufacturer’s instructions. Urinary albumin was assessed using an albumin ELISA (Alpha Diagnostic), according to the manufacturer’s instructions. Urinary IL-17 was assessed using a mouse IL-17 ELISA (R&D Systems), according to the manufacturer’s instructions.

### Preparation of cells for flow cytometry

#### Peripheral blood leukocytes

Blood was collected from the retroorbital plexus at the conclusion of the study. The blood was centrifuged at 350 × ***g*** for 5 min to isolate plasma. Erythrocytes were lysed by adding 10× volume of 1× PharmLyse (BD Biosciences, San Jose, CA). After incubation for 5 min at room temperature, the blood was centrifuged at 200 × ***g*** for 5 min. The pelleted peripheral blood leukocytes (PBL) were washed two times with 1× PBS, 2% FCS and centrifuged at 350 × ***g*** for 5 min.

#### Thymus

Thymocytes were isolated by pressing the thymus through a 70-micron cell strainer. Briefly, the filter was placed on a 50 ml of tube and wet with 5 ml of 1× PBS, 2% FCS. The thymus was then placed on the filter and pushed through the filter using a syringe plunger. The filter was subsequently washed with 15 ml of 1× PBS, 2% FCS. The cell suspension was centrifuged at 300 × ***g*** for 10 min and washed an additional time with 1× PBS, 2% FCS.

#### Bone marrow

For bone marrow isolation, tibias were harvested and cleaned of skin and muscle tissue. The ends of the bones were removed, and a 10 ml of syringe filled with Hank’s balanced salt solution (HBSS) and a 23 G needle was inserted into the end of the bone. The bone was flushed with 5 ml of HBSS on to a 70 micron filter over a 50 ml of tube. The same process was repeated on the other side of the bone, as well as with the other tibia. The cell suspension was centrifuged at 300 × ***g*** for 10 min and then washed an additional time with 10 ml of 1× PBS, 2% FCS.

#### Spleen

*S*pleens were homogenized using the Spleen Dissociation Kit (Miltenyi Biotec, Bergisch Gladbach, Germany) and the GentleMACS Octo Dissociator (Miltenyi Biotec), according to the manufacturer’s instructions.

#### Kidney

For isolation of renal immune cells, one kidney was homogenized in 5 ml of RPMI media containing 200 U/ml DNase and 10 mg/ml collagenase IV using the GentleMACS and a user-defined protocol for mouse kidney. The resulting homogenate was filtered through a 70 μM cell strainer and washed with 1× PBS containing 2% FCS and 2 mM EDTA. The single-cell suspension was centrifuged at 300 × ***g*** for 10 min.

Erythrocytes were lysed in 3 ml of 1× PharmLyse after the final washing step for 5 min at room temperature and washed with 15 ml of 1× PBS, 2% FCS. After centrifugation at 350 × ***g*** for 5 min, cells were used for flow cytometric analyses.

### Flow cytometric analyses

Briefly, cells were washed and resuspended in 1× PBS, 2% FCS, and 0.9% sodium azide at a concentration of 1 × 10^7^ cells/ml. A total of 1 × 10^6^ cells (100 μl) were aliquoted into a flow cytometry tube and incubated with 0.25 μg of anti-mouse CD32/CD16 (FcR block, BD Biosciences) for 5 min on ice. Cells were then stained with either isotype control antibodies or antibodies, shown in Supplementary Table S1, for 30 min on ice protected from light. All antibodies were diluted 1:200 in 1× PBS containing 2% FCS and 0.09% sodium azide. All antibodies utilized for staining in these experiments have been extensively validated by the company (BD Biosciences). When possible, a positive and negative control staining sample are included to further confirm specificity. Samples were analyzed on an LSR II or FACSymphony A3 flow cytometer (BD Biosciences), and a total of 50,000 CD45^+^ cells were acquired for each sample. Data were analyzed using FCS Express 7 Software (De Novo Software) or FlowJo version 10.8.2. Example gating strategies are in Supplementary Figures S1–3.

### Renal injury

Glomerulosclerosis was assessed in using hematoxylin and eosin staining of kidney sections as previously described by our laboratory [27]. To determine the glomerulosclerosis index, a total of 50 glomeruli were scored blinded using a scale of 0–4: 0: no sclerosis, 1: up to 25% of the glomerulus is sclerosed, 2: 26–50% of the glomerulus is sclerosed, 3: 51–75% of the glomerulus is sclerosed, 4: 76–50% of the glomerulus is sclerosed. The score was tabulated by multiplying each score by the number of glomeruli with that score divided by the total number scored.

### Statistical analysis

Data are presented as mean ± SEM. Statistical analyses were performed using GraphPad Prism 9. An unpaired *t*-test was used to analyze differences between control and SLE mice at baseline. An ordinary two-way ANOVA was used to analyze treatment (vehicle vs. leptin antagonist) or strain (NZW vs. NZBWF1) interactions. Multiple comparison testing was performed to analyze individual differences between treatment groups, and Tukey’s post-hoc test was used to correct for multiple comparisons. A *P-*value less than 0.05 is considered statistically significant.

## Results

**Figure 1:**
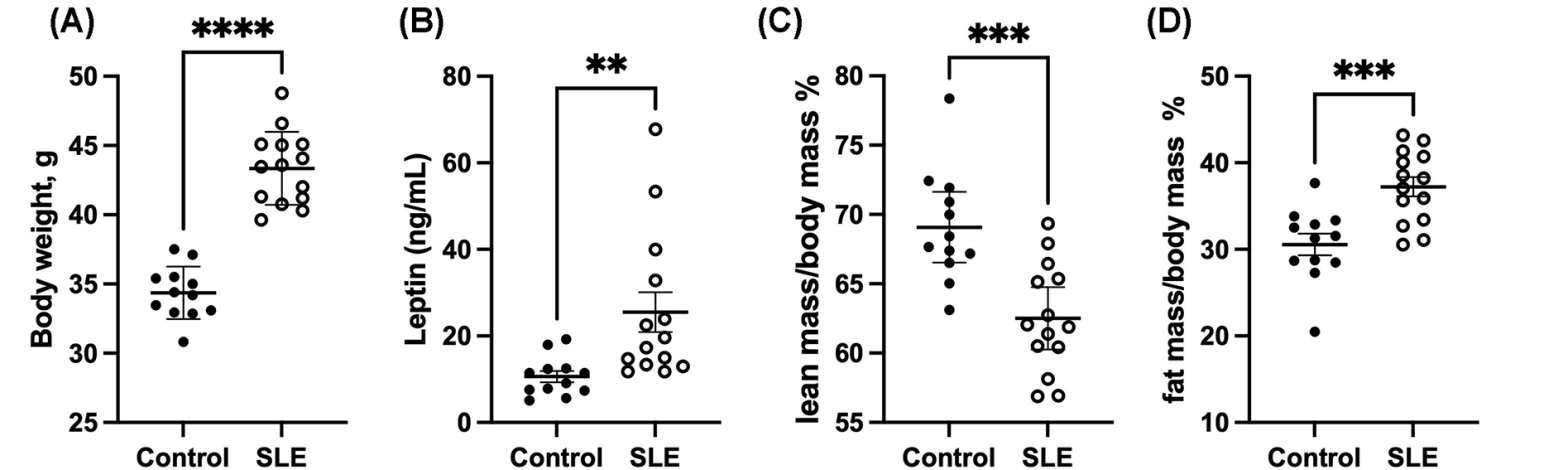
NZBWF1 (SLE) mice are obese and hyperleptinemic compared with control NZW (control) mice at 28 weeks of age (**A**) Fasting circulating leptin levels, as assessed by ELISA; (**B**) Body weight; (**C**) Lean mass/body mass (%) assessed using Echo MRI; (**D**) Fat mass/body mass (%) assessed using EchoMRI. **P<0.01, ***P<0.001, ****P<0.0001 by *t*-test. Animals per group: *n*=12 control, *n*=14 SLE.

**Figure 2:**
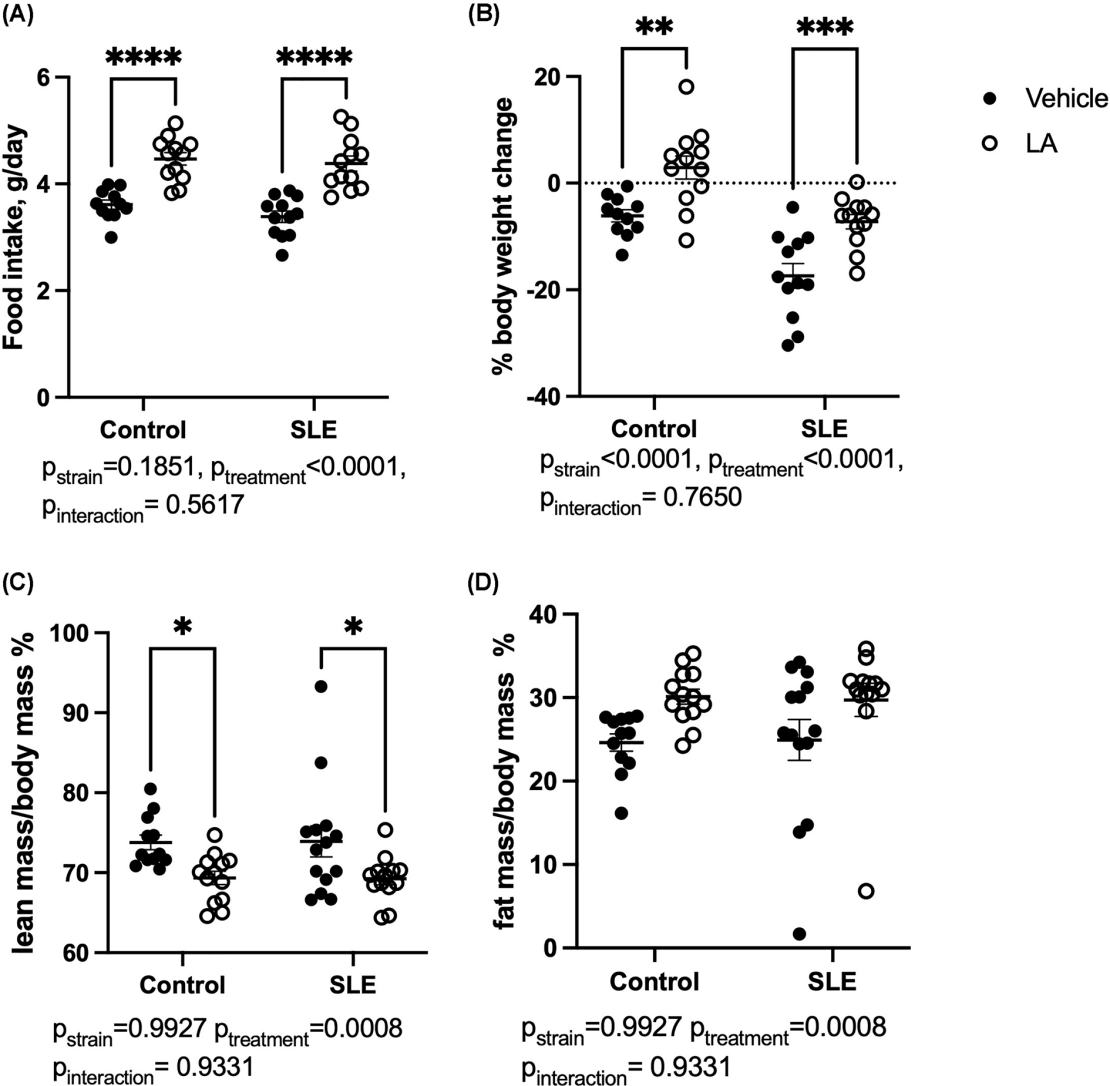
Effect of leptin antagonism on food intake and body composition (**A**) Average daily food intake for each individual mouse over the treatment period. (**B**) Percent change in body weight at the conclusion of the study. (**C**) Lean mass/body mass (%) assessed using Echo MRI. (**D**) Fat mass/body mass (%) assessed using EchoMRI. *P<0.05, **P<0.01 by two-way ANOVA followed by Tukey’s post-hoc test to determine significance. Animals per group: *n*=11, Control-vehicle; *n*=12, Control-LA; *n*=12, SLE-vehicle; *n*=12, SLE-LA.

SLE mice have higher body weight (Figure 1A, Control: 34.4 ± 0.5 vs. SLE: 43.4 ± 0.7 g, *P*<0.0001) and elevated circulating leptin levels (Figure 1B Control: 10.6 ± 1.3 vs. SLE: 25.5 ± 4.6 ng/ml, *P*=0.0078) compared with control mice at 28 weeks of age, in agreement with previously published data [28]. Echo MRI analyses indicate that the increased body weight is due to an increased fat mass, which is shown by increased fat mass/body mass percentage and decreased lean mass/body mass percentage (Figure 1C,D). To test the hypothesis that leptin plays a pathogenic role in the development of hypertension in SLE, control and SLE mice were treated with vehicle or LA via IP injection every other day for 4 weeks. Food intake was monitored daily over the course of the study (Figure 2A). Treatment with the leptin antagonist increased average daily food intake in both NZW and NZBWF1 animals (Control-vehicle: 3.6 ± 0.1 vs. Control-LA: 4.5 ± 0.1 g/day, *P*=0.006; SLE-vehicle: 3.4 ± 0.1 vs. SLE-LA: 4.5 ± 0.1 g/day, *P*<0.0001). There was no impact of LA treatment on fasting blood glucose in either control or SLE mice (Supplementary Figure S4). Body mass was also monitored over the course of the study (Figure 2B), and control mice administered LA had a significant increase in body weight during the treatment period (Control-vehicle: −5.8 ± 1.1% vs. Control-LA: 3.2 ± 2.0%, *P*=0.0011). SLE-vehicle mice lost weight during the treatment period, due to advanced SLE disease progression which leads to weight loss; however, this weight loss was attenuated in the mice that received LA (SLE-vehicle: −17.7 ± 1.9% vs. SLE-LA: −7.235 ± 1.36%, *P*=0.0001). The increase in body weight (or decrease in weight loss) is due to increased fat mass in both control and SLE mice (Figure 2C,D, p_treatment_ = 0.0008).

**Figure 3:**
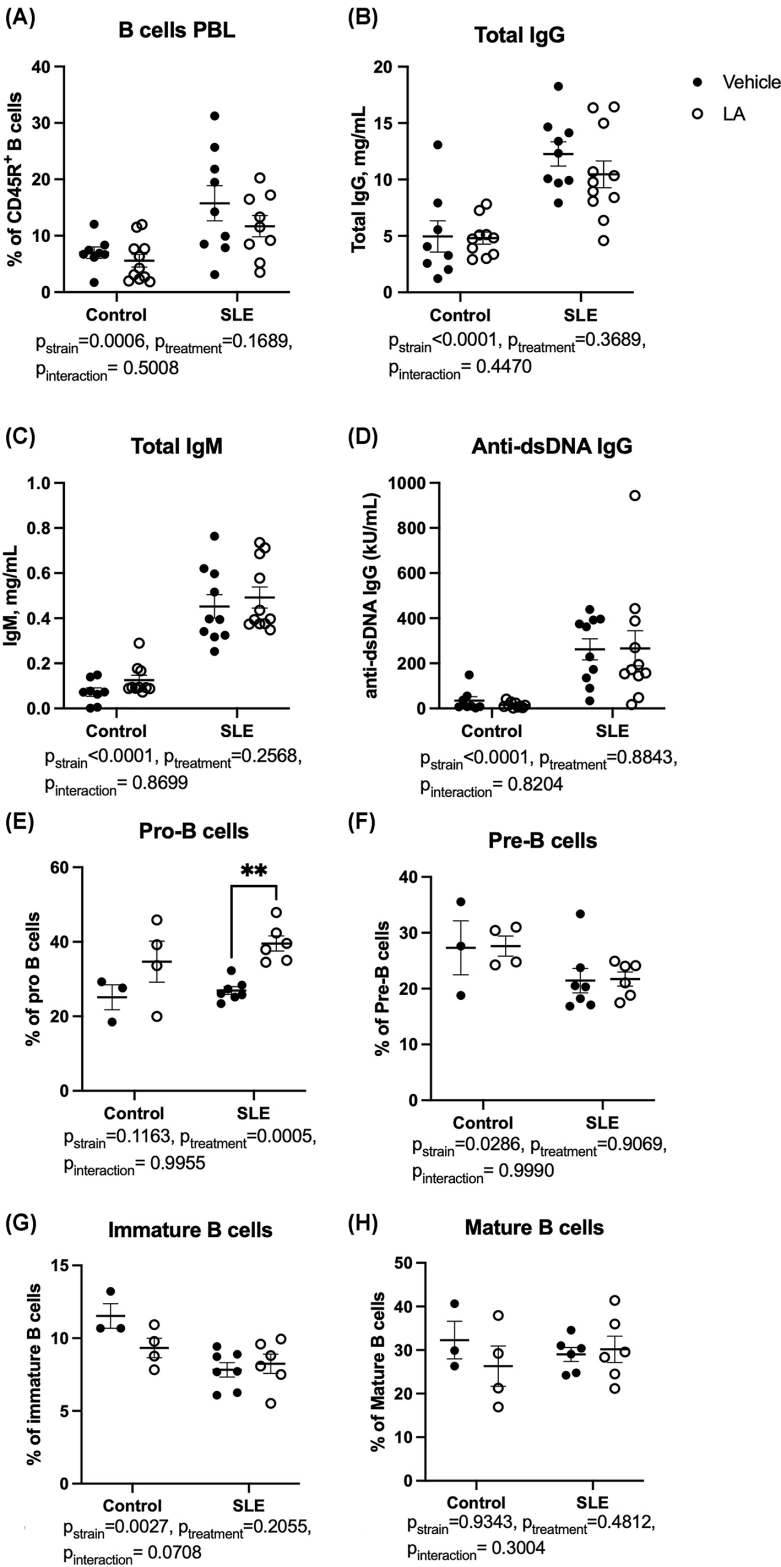
Effect of leptin antagonism on B cells, antibodies, and bone marrow B cell development (**A**) Circulating CD45R^+^ B-cell percentages as assessed using flow cytometry; (**B**) Total IgG; (**C**) Total IgM; (**D**) Anti-dsDNA IgG; (**E**) Percentage of pro-B cells (CD43^+^IgM^−^); (**F**) Percentage of pre-B cells (CD43^−^IgM^−^); (**G**) Percentage of immature B cells (IgM+IgD^low^); (**H**) Percentage of mature B cells (IgM^+^IgD^high^). **P<0.01 by two-way ANOVA followed by Tukey’s post-hoc test to determine significance. (A–D) Animals per group: *n*=8, Control-vehicle; *n*=10, Control-LA; *n*=10, SLE-vehicle; *n*=10 SLE-LA. (E–H) Animals per group: *n*=3, Control-vehicle; *n*=4, Control-LA; *n*=7, SLE-vehicle; *n*=6 SLE-LA.

**Figure 4:**
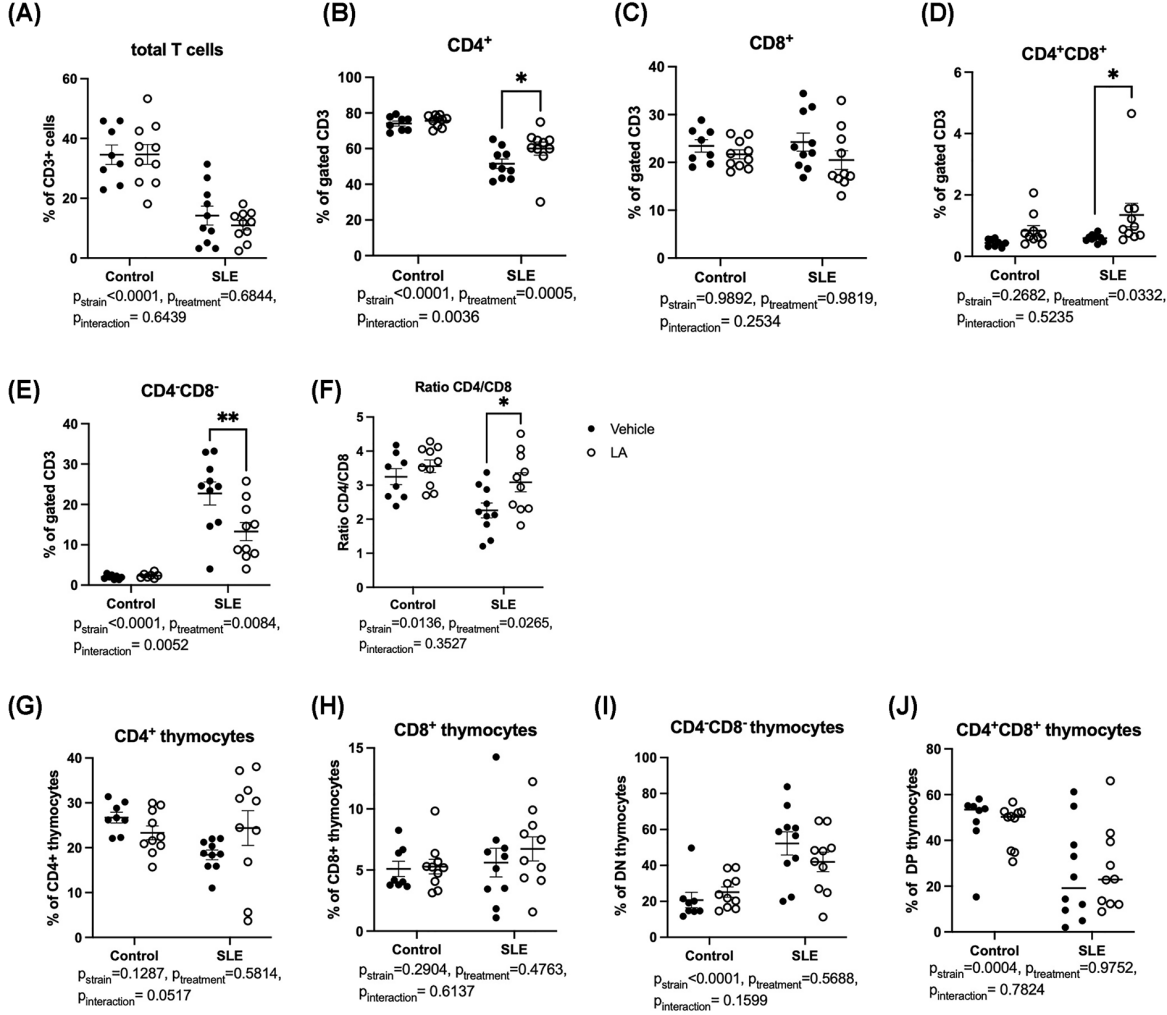
Leptin antagonism decreases circulating CD3^+^CD4^−^CD8^−^ (DN) T cells (**A**) Percentage of total CD3+ T cells; (**B**) Percentage of CD4+ T cells; (**C**) Percentage of CD8+ T cells; (**D**) Percentage of CD4+CD8+ T cells; (**E**) Percentage of CD4-CD8- T cells; (**F**) Ratio of CD4/CD8 T cells in peripheral blood; (**G**) Percentage of CD4^+^ thymocytes; (**H**) Percentage of CD8^+^ thymocytes; (**I**) Percentage of CD4^+^CD8^+^ thymocytes; (**J**) Percentage of CD4^−^CD8^−^ thymocytes. *P<0.05; **P<0.01, by two-way ANOVA followed by Tukey’s post-hoc test to determine significance. Animals per group: *n*=8, Control-vehicle; *n*=10, Control-LA; *n*=10, SLE-vehicle; *n*=10, SLE-LA.

We next analyzed circulating immune cell populations to assess the effect of LA on SLE disease activity. CD45R^+^ B lymphocyte percentages were elevated in SLE mice (Figure 3A, *P*_strain_ = 0.0006), but LA treatment did not change B-cell percentages. Similarly, SLE mice have elevated circulating IgG, IgM, and anti-dsDNA IgG compared with control mice, but none of these were lowered by treatment with LA (Figure 3B–D). We also considered whether B-cell development could be impacted in the bone marrow due to treatment with LA (Figure 3E–H). Bone marrow was harvested from the tibias of a subset of animals, and analyzed for the percentages of pro-B cells, pre-B cells, immature naïve B cells, and mature naïve B cells, according to the method of Claycombe et al. [23]. There were significant differences in the percentages of pre-B cells (Figure 3F, *P*_strain_ = 0.0286) and immature B cells (Figure 3G, *P*_strain_ = 0.0027) between control and SLE mice. Treatment with LA increased pro B cells in SLE mice (Figure 3E, *P*<0.001). Circulating T cells were also examined (Figure 4). The percentages of total CD3^+^ T cells were not impacted by treatment with LA, although SLE mice had significantly lower circulating CD3^+^ T cells (Figure 4A, *P*_strain_<0.0001), as previously shown by our laboratory [38,39]. When analyzing the proportions of CD4^+^, CD8^+^, CD4^+^CD8^+^ (DP), and CD4^−^CD8^−^ (DN) T cells (Figure 4B–E), there were significant differences between control and SLE mice in the percentages of DN T cells and CD4+ T cells (DN T cells: *P*_strain_<0.0001, CD4^+^ T cells: *P*_strain_<0.0001). SLE mice treated with leptin antagonist had an increase in the percentage of CD4+ T cells (49.9 ± 2.7% of total CD3 vs. 63.3 ± 1.8 of total CD3 and a significant decrease in the percentage of DN T cells (SLE-vehicle: 23.8 ± 2.2% of total CD3 vs. SLE-LA: 13.2 ± 2.5%, of total CD3, *P*=0.0004). The shifts in the T*-*cell populations led to an increase in the CD4/CD8 ratio in SLE mice (Figure 4F; SLE-vehicle: 2.3 ± 0.8 vs. 3.2 ± 0.9, *P*=0.046). When analyzing T-cell development in the thymus (Figure 4G–J), we found similar trends in T-cell subsets, including increased DN thymocytes *(P*_strain_<0.0001) and decreased DP thymocytes (*P*_strain_=0.0004) in SLE compared with controls. Percentages of circulating neutrophils and monocytes were also analyzed, and there were no significant changes due to LA treatment (Supplementary Figure S5).

**Figure 5:**
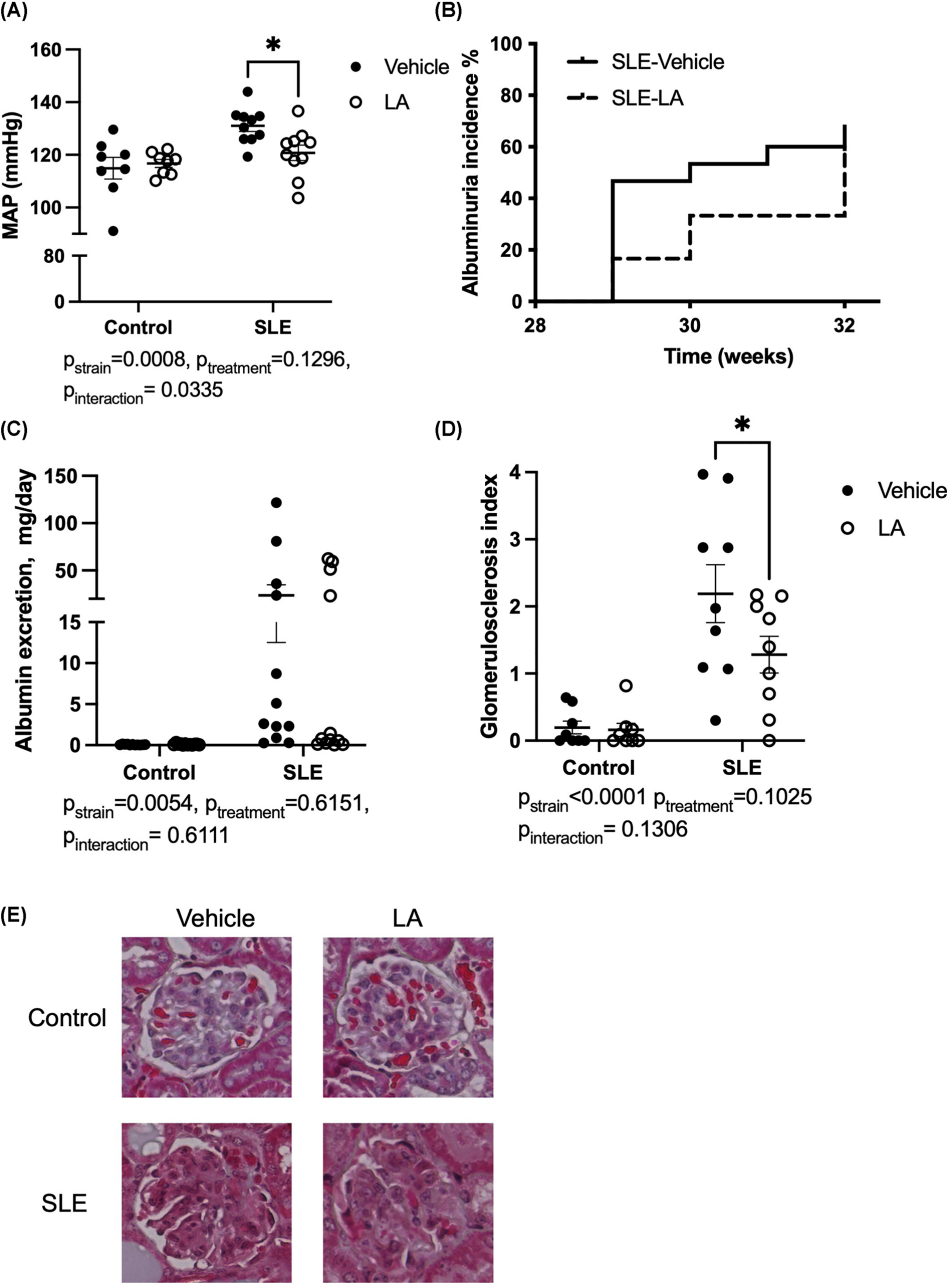
Leptin antagonism lowers blood pressure and ameliorates glomerular injury (**A**) Mean arterial pressure, as assessed by carotid artery catheter in conscious freely moving mice. (**B**) Albuminuria incidence, assessed weekly using dipstick analyses of overnight urine. (**C**) Albumin excretion rate, as assessed by ELISA; (**D**) Glomerulosclerosis index; (**E**) Representative images of glomerulosclerosis (20×) from paraffin-embedded kidneys stained with H&E. *P<0.05 by two-way ANOVA followed by Tukey’s post-hoc test to determine significance. Animals per group (A) *n*=8, Control-Vehicle; *n*=8, Control-LA; *n*=11, SLE-vehicle; *n*=10 SLE-LA. (B,C) *n*=11, Control-vehicle; *n*=12, Control-LA; *n*=12, SLE-vehicle; *n*=12, SLE-LA; (**D**) *n* = 9 per group.

**Figure 6:**
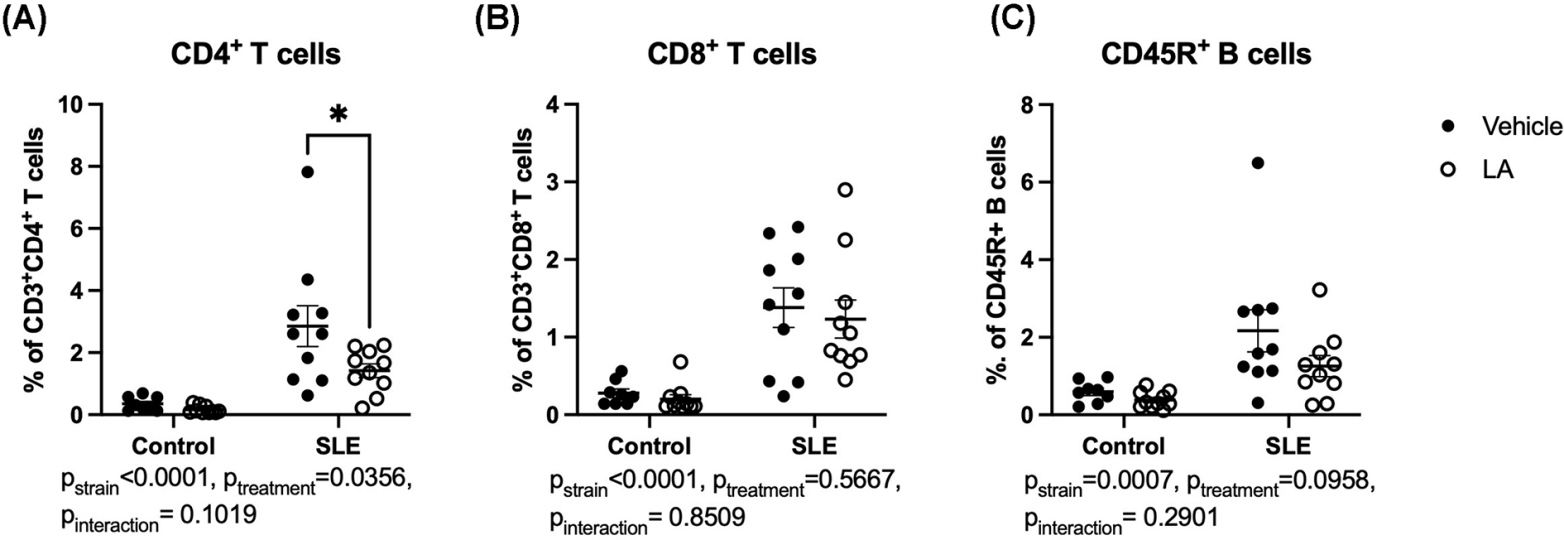
Leptin antagonism decreases renal immune cell infiltration (**A**) Percentage of renal CD4^+^ T cells; (**B**) Percentage of renal CD8^+^ T cells; (**C**) Percentage of renal CD45R^+^ B cells. *P<0.05 by two-way ANOVA followed by Tukey’s post-hoc test to determine significance. Animals per group: *n*=8, Control-vehicle; *n*=10, Control-LA; *n*=10 SLE-vehicle; *n*=10 SLE-LA.

At the conclusion of the study, carotid artery catheters were implanted, and blood pressure was measured on two consecutive days (Figure 5A). SLE mice have elevated blood pressure compared with control mice (Control-vehicle: 114 ± 4 vs. SLE-vehicle: 131 ± 2 mmHg, *P*_strain_ = 0.0012), as previously shown by our laboratory and others. Treatment with leptin antagonist had no impact on blood pressure in control animals; however, SLE mice administered LA had decreased BP (SLE-LA: 121 ± 3 mmHg, *P*=0.02). We also analyzed the impact of leptin antagonism on renal injury. SLE mice administered LA had a slightly delayed albuminuria development, as assessed by weekly dipstick analyses of overnight urine collection (Figure 5B). The albumin excretion was also quantified at the conclusion of the study (Figure 5C). SLE mice have increased albumin excretion compared with controls (*P*_strain_ = 0.0054), but treatment with LA did not significantly impact albumin excretion in SLE mice at the conclusion of the study. Glomerulosclerosis was assessed in H&E-stained sections (Figure 5D,E). SLE mice have increased glomerular injury compared with controls (*P*_strain_<0.0001), and there is a decrease in injury in SLE mice treated with LA (SLE-vehicle: 2.2 ± 0.4 vs. SLE-LA 1.3 ± 0.3, *P*=0.049). Finally, we analyzed the impact of leptin antagonist treatment on renal immune cell infiltration (Figure 6). SLE mice have increased renal B and T lymphocytes compared with control mice (B cells: *P*_strain_ = 0.0053, CD4 T cells: *P*_strain_<0.0001, and CD8 T cells: *P*_strain_<0.0001). Treatment with LA lowered renal CD4^+^ T cell percentages (SLE-vehicle: 2.7 ± 0.65 vs. SLE-LA: 1.3 ± 0.2%, *P*=0.02). SLE mice treated with LA had lower urinary IL-17, suggesting that renal IL-17 was lower due to the reduced T-cell infiltration (Supplementary Figure S6).

## Discussion

Since its discovery in 1994 [41], the biological role of leptin has been intensely studied, and the adipokine is now appreciated as an central mediator that impacts myriad physiological processes, including appetite, metabolic status, energy expenditure, glucose homeostasis, reproduction, angiogenesis, and immune responses, among others. These studies have also led to a greater understanding of the link between metabolism and immunity. In the present study, we tested the hypothesis that elevated leptin plays a pathogenic role in SLE disease progression and promotes the development of autoimmune-associated hypertension and renal injury in the NZBWF1 mouse model of SLE, which has elevated leptin levels. The major findings of this study are as follows: Treatment with a PEGylated leptin antagonist (1) increases food intake and prevents cachexia in SLE mice, (2) decreases DN T cells in the circulation and increases the CD4/CD8 ratio, (3) lowers blood pressure, and (4) decreases renal glomerular injury and renal immune cell infiltration.

Hyperleptinemia is associated with elevated circulating levels of various inflammatory mediators in both animal models and individuals with obesity. In addition, elevated leptin levels are associated with leptin resistance, or decreased responsiveness to the central effects of leptin to control appetite [42]. While this concept is incompletely understood, several mechanisms have been proposed, including saturation in the transport of leptin across the BBB [43–45], hypothalamic low-grade inflammation that impairs leptin signaling [46], direct interaction of C-reactive protein with leptin [47], and LepR degradation by matrix metalloproteinase-2 in the hypothalamus of obese rodents [48]. It should be noted, however, that a recent study determined that lean and diet-induced obese mice similarly accumulate leptin in brain areas that control appetite and energy expenditure, suggesting that BBB transport is not impaired [49]. C57BL/6 mice have leptin levels of approximately 5–15 ng/ml depending on age and sex [50,51], similar to NZW mice in the present study, who have an average leptin concentration of 10.6 ng/ml. Conversely, SLE mice on a normal chow diet have an average of 25.5 ng/ml (Figure 1). The leptin antagonist used in the present study can cross the BBB and has been detected in the CNS after administration peripherally [52]. Previous studies in male and female C57BL/6 mice fed normal chow reported an approximately 40% increase in food intake and a 20% increase in body weight after treatment with LA for approximately a week [52] or 1 month [53]. In the present study, we saw more modest increases in body weight (9% in control and 10.5% in SLE) and food intake (∼20%) in both control and SLE mice (Figure 2). Notably, these results are analogous to a study in which mice with chronic kidney disease were given the same antagonist [53]. While not directly tested in the present study, this suggests that there may some degree of central leptin resistance, as it seems that LA is less effective in increasing food intake in the NZW and NZBWF1 mice used in the present study when compared with C57BL/6 mice used in other studies. Central resistance to treatment with a leptin antagonist has been shown using a similar compound. Hypothalamic leptin resistance was induced by giving a ICV injection of an AAV encoding leptin. In this case, the rats initially consume fewer calories, but gradually increase food intake and body weight to levels similar to controls. Thus, they develop leptin resistance in the absence of obesity. In response to leptin antagonism, the control rats had a significant increase in food intake in response to the antagonist, while the leptin resistant rats only had a slight increase in food intake [54]. Nevertheless, the results in the present study indicate that an assessment of resistance to the central effects of leptin in SLE will require further investigation.

A multitude of studies have shown that leptin is critical for optimal innate and adaptive immune function [55]. Most immune cells express the long form of the leptin receptor (ObRb) and respond directly to leptin via receptor binding. The downstream signaling predominately mediates proinflammatory activity that is important for infection clearance such as promoting neutrophil chemotaxis, macrophage phagocytosis, and NK cell cytotoxic activity [56]. Initial observations in *ob/ob* and *db/db* mice, which lack leptin or leptin receptors, respectively, revealed that defects in leptin signaling lead to impaired immunity, including reduced antibody responses, and decreased cytotoxic responses [57,58]. Subsequently, studies in animal models of autoimmunity showed that leptin promotes autoimmune disease progression and allergic responses [24–27,59]. These results are echoed in humans; individuals with a mutation in leptin or the leptin receptor have increased risk of infection due to immunodeficiency [13,60]. Leptin antagonism led to several immunological changes in SLE mice, including an increase in the percentage of circulating CD4^+^ T cells, an increase in the CD4/CD8 ratio, and a decrease in circulating DN T cells. Lower percentages of CD4 T cells, and a lower CD4/CD8 ratio in the peripheral blood, are common in murine SLE models [61], and in patients with SLE [62,63]. An improvement in this ratio (higher CD4/CD8) has previously been used as an indicator of improvement in disease manifestations in response to therapy such as cyclosporine [63]. Based on the results in the present study, it is unclear whether the effect on CD4 T cells and the CD4/CD8 ratio is due to decreased binding of the leptin receptor to T cells in the periphery, changes in T-cell development/maturation, or secondary to the effects of LA on other cells or tissues. There was a strong trend for an increase in CD4^+^ thymocytes in the SLE mice that received the leptin antagonist (Figure 4G), so it may be that positive selection of DP thymocytes into a CD4^+^ or CD8^+^ thymocytes is impacted in some way.

In SLE patients and murine models of the disease, DN T cells are substantially increased in the circulation [64]. In the present work, we similarly found increased DN T cells in the peripheral blood of SLE mice, which were reduced by leptin antagonism. DN T cells express either the αβ or γδ T cell receptor and are typically a small population of cells in in the peripheral blood (3–5%) [65]. They function in seemingly contradictory roles, from induction of peripheral tolerance and disease prevention to playing a role in end organ damage in various inflammatory diseases [66]. In patients with SLE, DN αβ T cells are a significant source of IL-17 and IFN-γ and they also infiltrate the kidneys [64]. The origin of DN T cells is not completely clear, although several studies have suggested that they arise from activated CD8^+^ T cells [67,68]. In addition, DN T cells in the periphery may be the result of an escape of negative selection in the thymus and subsequent activation and expansion in the periphery [69,70]. Based on the data from the present study, it is unclear whether the DN T cells are derived from CD8^+^ T cells in the periphery or escape from thymic selection. There was a trend for a decrease in DN T cells in the thymus of SLE mice treated with LA (Figure 4I), again suggesting that T-cell development may have been impacted by leptin antagonist treatment. Leptin can be an important regulator of thymopoiesis; *ob/ob* mice exhibit thymic atrophy [71]. In addition, several studies have suggested that leptin provides a stimulatory role for T-cell development in situations of acute thymic atrophy, such as starvation [71], immunosuppressive therapy [72], or infection [73]; but leptin administration does not augment thymopoiesis in normal (non-obese) mice [74]. Finally, leptin receptor expression has been found on DN, DP, and CD4+ thymocytes, but not CD8+ thymocytes [75], further supporting a role for leptin antagonism impacting T-cell development.

Leptin antagonism significantly lowered blood pressure in SLE mice. The role of leptin in blood pressure control is complex, as leptin acts centrally in the hypothalamus to increase SNS activity [9,10] but can also relax arteries via endothelial cell leptin receptor-mediated increases in nitric oxide (NO) [76]. Initial studies to understand these seemingly paradoxical actions found that when animals were treated with the NO synthase inhibitor L-NAME, animals had an exacerbation of their BP response to leptin, which led to the theory that leptin derived-NO production counteracts the sympatho-activatory effects of leptin [77,78]. However, a recent study that utilized a mouse with endothelial cell-specific deletion of the leptin receptor found that endothelial leptin signaling does not contribute to leptin’s ability to increase blood pressure [79]. Importantly, all the previously discussed studies were conducted in male animals, and elevated leptin levels are not associated with increased sympathetic activation in females [80,81]. Huby et al. demonstrated that leptin increases BP via aldosterone-dependent mechanisms in female mice, namely by inducing endothelial dysfunction [82]. While we did not test endothelial function in the present study, NZBWF1 mice do develop endothelial dysfunction as disease progresses [29]. In a previous study, Mathis et al. tested the role of renal sympathetic nervous system activity in the NZBWF1 mouse and found that renal denervation did not change blood pressure. Together, the data from Mathis et al. and the present study suggest that leptin antagonism does not lower blood pressure because of changes in renal sympathetics.

Finally, treatment with LA improved renal injury in SLE mice (Figures 5 and 6). Leptin receptors (both the long form ObRb and to a greater extent, ObRa) are expressed in the kidney. Previous studies showed that binding of leptin within the kidney is primarily within the renal medulla, most likely in the vasculature [83]. The binding of leptin to renal endothelial cells leads to TGF-β production, which can further promote collagen production by mesangial cells, leading to fibrosis and glomerulosclerosis [84]. Thus, leptin antagonism may directly protect from the development of glomerulosclerosis and renal injury. In addition, T cell-specific leptin receptor signaling may directly impact CD4^+^ T cell trafficking or effector function within the kidney. Finally, CD4^+^ T cells are an important source of cytokines such as TNF-α, IFN-γ, and IL-17 within the kidney, which promote renal inflammation. Specifically, TNF-α, which is secreted by CD4+ T_H_ cells, has been shown to decrease renal blood flow and GFR in mice [85], which may be due to increased reactive oxygen species and thromboxane [85,86]. IL-17 can promote renal fibrosis and tissue inflammation as well as induce neutrophil infiltration [87]. The proinflammatory milieu has been demonstrated to be involved in the pathogenesis of renal damage by altering renal vascular function and fluid electrolyte balance [88], increasing renal oxidative stress [89], and altering sodium reabsorption by renal tubules [90]. While there are limited data on the renal effects of leptin antagonism, a very recent study in pigs showed that antagonism of leptin prevents inflammation in a porcine model of ischemia reperfusion [91]. As we have previously published, the rates of albuminuria vary in this model of SLE [37,39,92], and treatment with LA did not improve albumin excretion despite improving glomerular injury (Figure 5).

In conclusion, the present study demonstrated the immunomodulatory and physiological impact of leptin antagonism in a clinically relevant mouse model of SLE. The present study has several limitations. First, because the leptin antagonist crosses the blood brain barrier, it is currently unknown whether the effects of the treatment are via central or peripheral effects. Second, we did not assess SNS activity or endothelial function to further characterize the reasons for changes in BP in SLE mice. However, the data presented here suggest that leptin plays a role in SLE disease progression in a mouse model that is obese and has elevated leptin levels, similar to the SLE patient population.

## Clinical perspectives

- The autoimmune disease systemic lupus erythematosus (SLE) is characterized by imbalances in various cytokines, including the adipokine leptin, which may play a role in immune system dysfunction and the development of hypertension.
- Control and SLE mice were treated with a leptin antagonist, which decreased circulating pathogenic DN T cells, lowered blood pressure, and decreased renal injury in SLE mice.
- These data suggest that leptin plays a pathogenic role in SLE disease progression, and that therapies that target leptin signaling may be beneficial in some SLE patients.

## Supplementary Material

Supplementary Figures S1-S6 and Table S1

## Data Availability

All materials, data, and protocols are present in the manuscript or are available upon request.

## Open Access

Open access for this article was enabled by the participation of University of Mississippi in an all-inclusive *Read & Publish* agreement with Portland Press and the Biochemical Society under a transformative agreement with Individual.

## Abbreviations

DN: double negative
DP: double positive
BBB: blood brain barrier
GFR: glomerular filtration rate
IFN: interferon
IL: interleukin
LEPR: leptin receptor
LA: leptin superantagonist
L-NAME: N(gamma)-nitro-L-arginine methyl ester
NO: nitric oxide
SLE: systemic lupus erythematosus
SNS: sympathetic nervous system
TGF: transforming growth factor
Th: T helper
TNF: tumor necrosis factor
