# Supplementary material for: Leptin antagonism attenuates hypertension and renal injury in an experimental model of autoimmune disease
Source: Clin Sci (Lond). 2023 Dec 14;137(23):1771–85. doi: 10.1042/CS20230924 (PMC10721433; doi:10.1042/CS20230924)
Supplement: Supplementary Figures S1-S6 and Table S1 [file CS-2023-0924_supp.pdf]

Table S1. Monoclonal antibodies used for flow cytometry.

Antibody	Supplier	Clone
CD32	BD Biosciences	2.4G2
CD45	BD Biosciences	30-F11
CD3	BD Biosciences	145-2C11
CD4	BD Biosciences	GK1.5
CD8α	BD Biosciences	53-6.7
CD45R	BD Biosciences	RA3-6B2
Ly6G	BD Biosciences	1A8
Ly6C	BD Biosciences	RB6-8C5
CD11b	BD Biosciences	M1/70

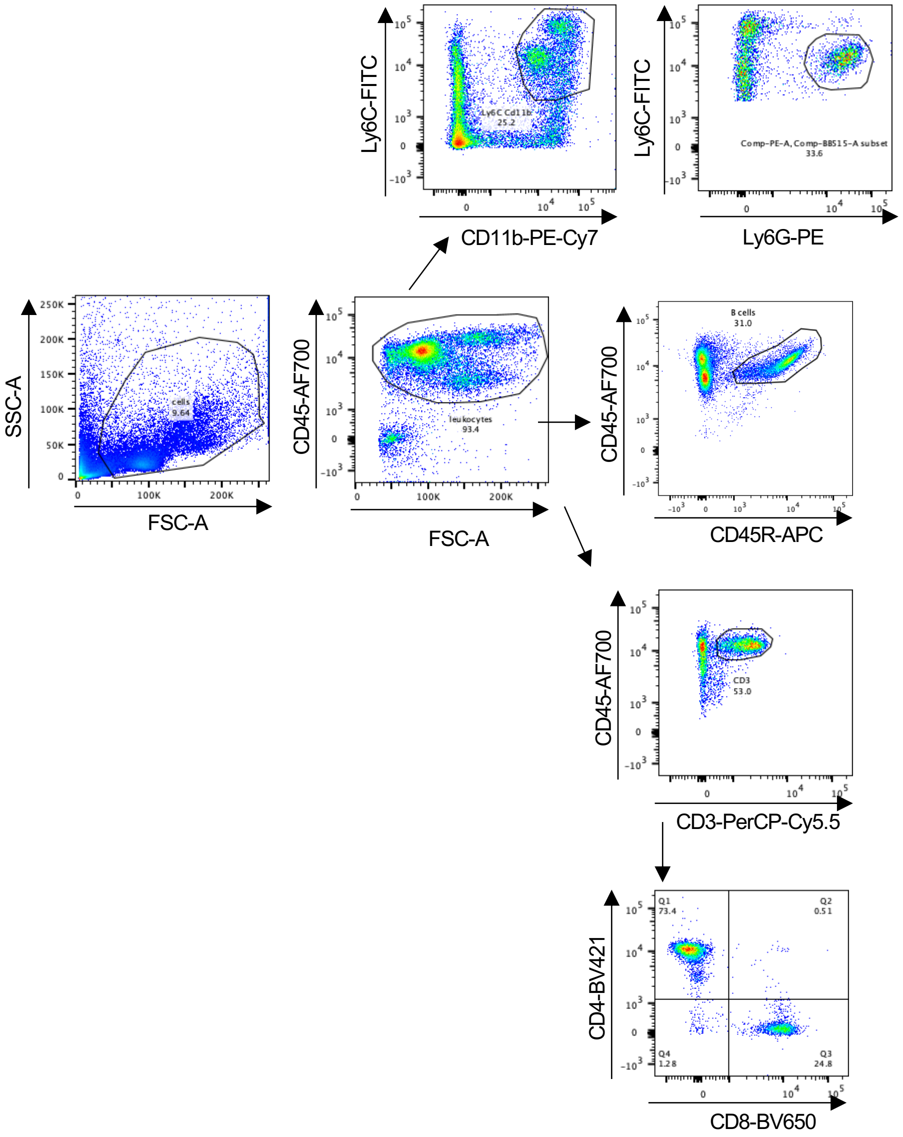

Figure S1. Gating Strategy to identify peripheral blood immune cell populations.

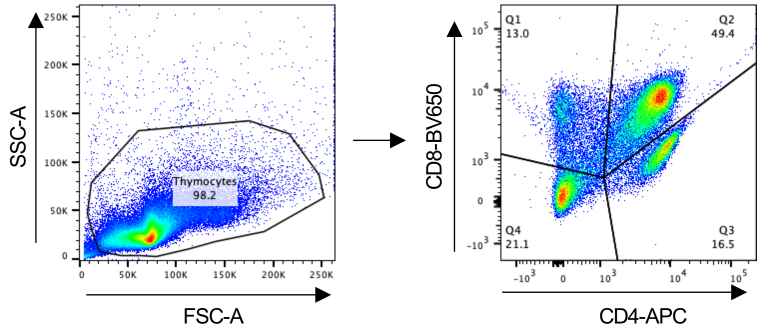

Figure S2. Gating strategies to characterize thymocytes.

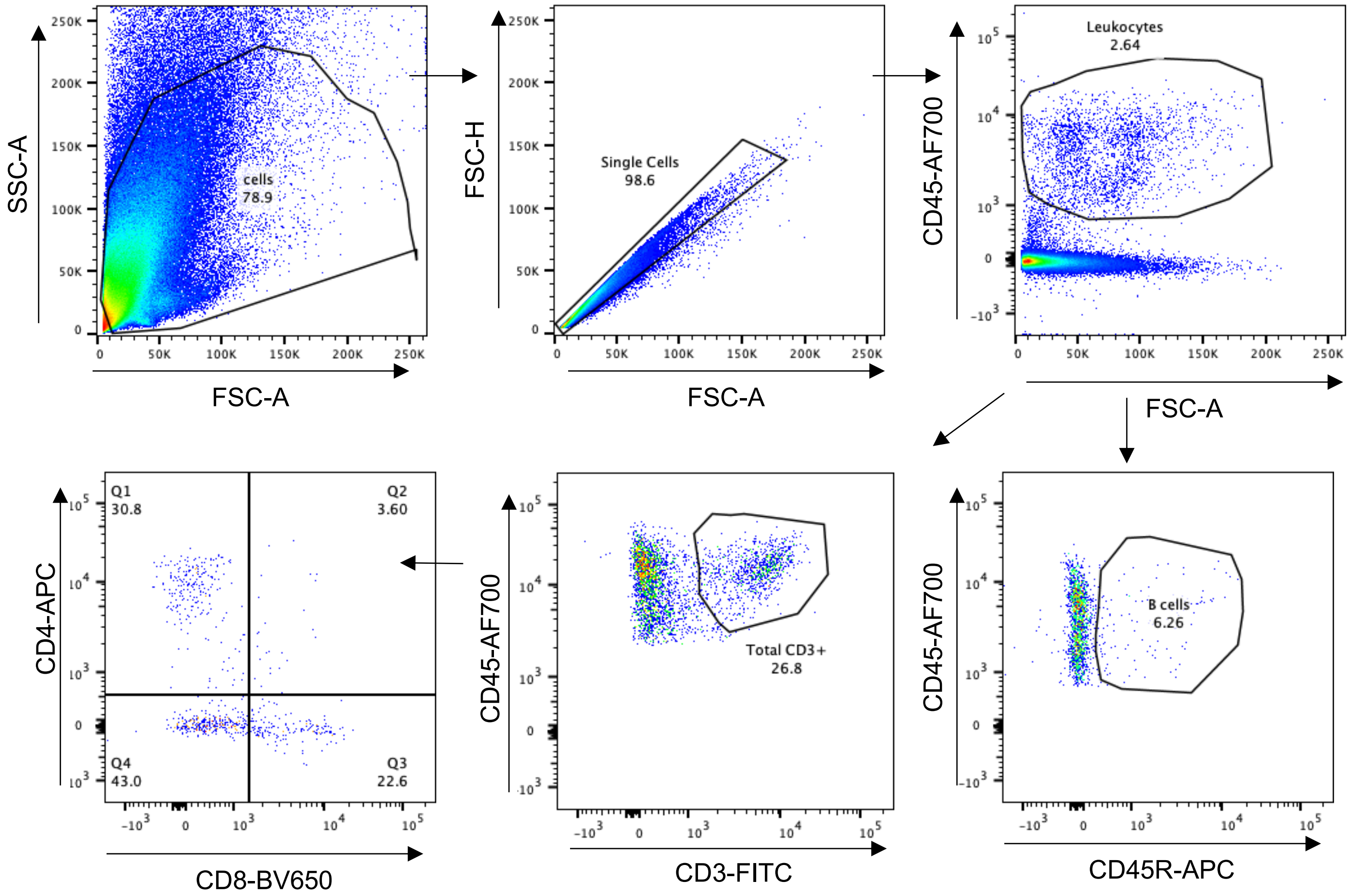

Figure S3. Gating strategy to characterize renal immune cells.

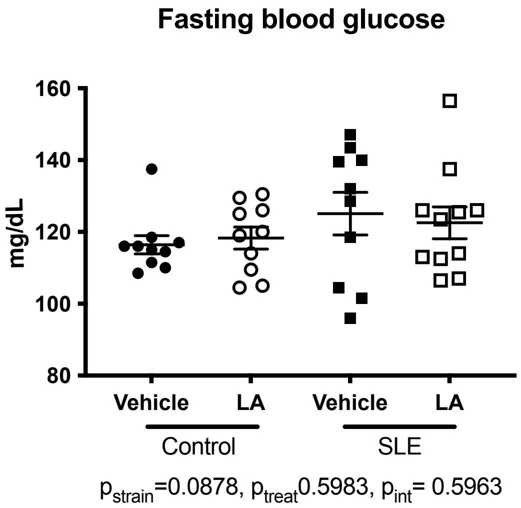

Figure S4. Fasting blood glucose in control and SLE mice treated with vehicle or leptin antagonist. Mice were fasted for 5 hours, and blood was sampled from the tail. Blood glucose was tested using an AccuCheck Glide glucometer Each mouse was tested twice.

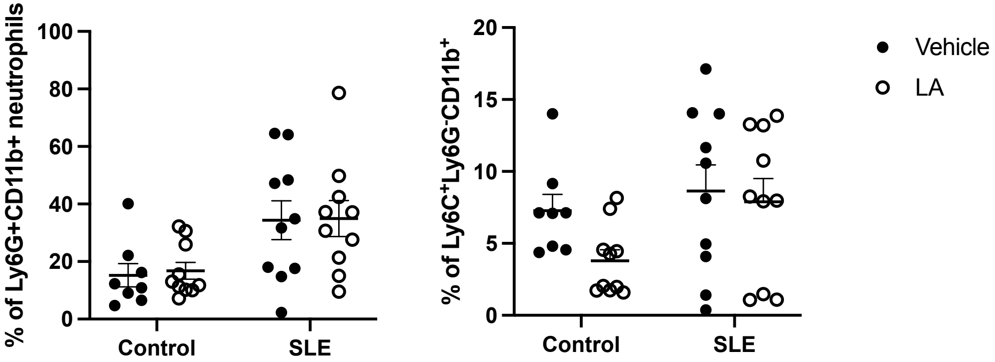

p_{strain}=0.0015, $p_{treatment}$=0.9439, $p_{interaction}$= 0.9299

p_{strain}=0.0651 $p_{treatment}$=0.1486 $p_{interaction}$= 0.3469

Figure S5. A: Percentage of neutrophils, B: Percentage of monocytes in peripheral blood at the conclusion of the study.

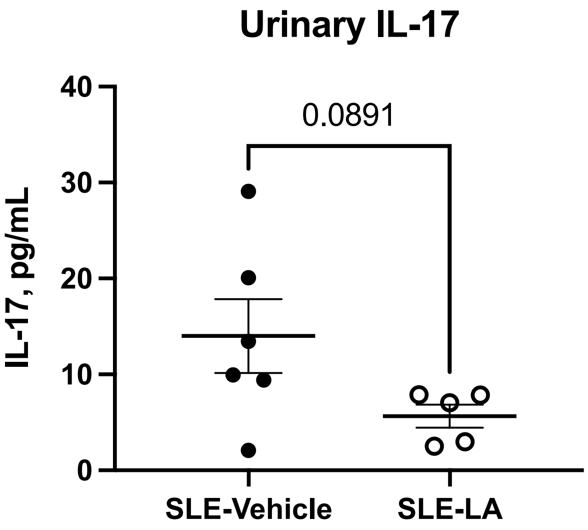

Figure S6. Urinary IL-17, as assessed by ELISA, in SLE-vehicle and SLE-LA mice at the conclusion of the study.
